# Monkeypox Content on TikTok: Cross-sectional Analysis

**DOI:** 10.2196/44697

**Published:** 2023-01-17

**Authors:** Antonio Ji-Xu, Kyaw Zin Htet, Kieron S Leslie

**Affiliations:** 1 Department of Dermatology University of California, Davis Sacramento, CA United States; 2 Tulane University School of Medicine New Orleans, LA United States; 3 Department of Dermatology University of California, San Francisco San Francisco, CA United States

**Keywords:** TikTok, social media, monkeypox, mpox, pandemic, epidemic, infectious disease, outbreak, quality assessment, content analysis

## Introduction

Approximately 81,000 mpox (monkeypox) cases have been confirmed globally during the ongoing 2022 outbreak [1]. Online and social media platforms can help disseminate up-to-date information on mpox but can also cause patient harm by propagating misinformation [2,3]. TikTok is currently the most downloaded app globally, with >1 billion monthly active users [4]. Recently, TikTok expanded the duration of videos to 10 minutes, increasing its potential to disseminate information, similar to other platforms such as YouTube [5]. However, there have been few studies investigating the quality of mpox content on TikTok [6]. Hence, we analyzed trending mpox content on TikTok to assess metrics affecting user engagement and content quality.

## Methods

TikTok was searched for videos using the term “monkeypox” on August 27, 2022. The top videos returned by the TikTok search algorithm were analyzed. Videos unrelated to mpox, non-English videos, and duplicates were excluded. Video type, metrics, content, and content quality were collected and assessed by two independent reviewers. Content quality was assessed using DISCERN, a validated 16-item instrument to evaluate the quality of health information scored from 1 (poor) to 5 (excellent) [7]. For videos with no content on treatment options, the DISCERN section on treatment was excluded during scoring. Content accuracy was assessed using the Centers for Disease Control and Prevention mpox resource [1]. The identity of content creators was determined using biographic information on TikTok and through Google searches. Any disagreements were resolved by a dermatologist with an interest in mpox. Associations between hashtags and the number of views were evaluated with multivariable regression. Means between two groups were compared with student *t* test. A 2-sided *P* value <.05 was considered statistically significant. Statistical analyses were performed using R version 4.1.3 (R Foundation for Statistical Computing).

## Results

Overall, the top 137 videos were screened to include 100 videos: 55% (n=55) originated from health care professionals or scientists, 38% (n=38) from non–health care professionals or scientists, and 7% (n=7) from news organizations. Of the accounts from health care professionals or scientists, 65% (n=36) were physicians; 15% (n=8) were physician assistants, nurse practitioners, or nurses; 5% (n=3) were epidemiologists; and 15% (n=8) were other health care professionals or scientists. Included videos had a combined 306,035,200 views and 27,785,816 likes. Hashtags related to viruses and the lesbian, gay, bisexual, transgender, queer (LGBTQ+) community were associated with a higher number of views (both *P*<.001; Table 1).

The mean DISCERN score was 2.8 (SD 1.0), with high interrater reliability (Cohen κ>0.8). Physician videos had a significantly higher mean DISCERN score (4.0) and lesser variation in scores (SD 0.5) compared to nonphysician videos (DISCERN score mean 2.1, SD 1.0; *P*<.001). Low DISCERN scores were most common on items involving sources of information and treatment choices. Only one video mentioned treatment choices, discussing tecovirimat, and no other treatments were mentioned. Similarly, fewer of the 36 physician videos had misleading statements (n=5, 14%) compared to the 64 nonphysician videos (n=21, 33%). Nonetheless, videos from nonphysicians had a higher mean number of views and likes than videos from physicians (both *P*<.001). Videos with misleading statements also had more views (mean 4,820,885) compared to those without (mean 2,409,196; *P*<.001).

**Table 1:** Metrics of trending mpox content on TikTok (N=100).

		Videos, n (%)	Number of views, mean (SD)	Number of likes, mean (SD)	DISCERN score, mean (SD)	Misleading statement, n (%)
**Content creator**						
	Nonphysician	64 (64)	4,145,664 (3,094,378)	375,333 (187,666)	2.1 (1.0)	21 (33)
	Physician	36 (36)	1,130,908 (932,968)	104,570 (131,871)	4.0 (0.5)	5 (14)
**Video type**						
	Educational content	82 (82)	2,910,243 (2,652,819)	428,883 (214,441)	3.1 (0.8)	19 (23)
	Patient experience	72 (72)	4,957,425 (3,649,648)	337,284 (322,406)	2.4 (1.0)	6 (8)
	Entertainment	9 (9)	5,676,122 (3,536,727)	795,933 (550,651)	1.6 (1.1)	7 (78)
	News	5 (5)	1,704,400 (579,805)	288,498 (75,700)	3.8 (0.3)	0 (0)
**Hashtag categories**						
	Virus	26 (26)	4,368,069 (3,994,561)	325,704 (355,680)	2.5 (1.0)	6 (23)
	Outbreak	17 (17)	2,619,524 (1,790,431)	216,393 (236,373)	2.8 (0.9)	3 (18)
	Symptoms	15 (15)	1,273,793 (555,833)	83,170 (66,488)	2.8 (0.8)	4 (27)
	Vaccine	13 (13)	1,618,308 (1,445,424)	288,437 (292,527)	3.0 (0.9)	0 (0)
	LGBTQ+^a^	12 (12)	7,161,225 (5,645,119)	139,936 (119,348)	3.5 (0.6)	0 (0)

^a^LGBTQ+: lesbian, gay, bisexual, transgender, queer.

## Discussion

Our findings highlight that although physician-created content was of higher quality and contained fewer misleading statements, it had reduced engagement compared to non–physician-created content and rarely discussed management. Since tecovirimat is likely most effective when started early, education in this area could improve patient outcomes [1]. Limitations of our study include the cross-sectional design and that DISCERN was originally validated to assess written educational content. Owing to the lack of validated instruments for this purpose, DISCERN has recently been used by multiple studies to evaluate the quality of videos on social media [8,9]. Given the increasing use of social media for health information, there is a need for the development and validation of specific instruments for quality assessment. Physician creators posting mpox content on TikTok should consider including a discussion of treatments and using hashtags selectively to increase user engagement.

## Abbreviations

LGBTQ+: lesbian, gay, bisexual, transgender, queer.
